# Combination Strategies with HSP90 Inhibitors in Cancer Therapy: Mechanisms, Challenges, and Future Perspectives

**DOI:** 10.3390/ph18081083

**Published:** 2025-07-22

**Authors:** Yeongbeom Kim, Su Yeon Lim, Hyun-Ouk Kim, Suk-Jin Ha, Jeong-Ann Park, Young-Wook Won, Sehyun Chae, Kwang Suk Lim

**Affiliations:** 1 Department of Smart Health Science and Technology, Kangwon National University, Chuncheon 24341, Republic of Korea; kyb8234@kangwon.ac.kr (Y.K.); suyeonlim9846@kangwon.ac.kr (S.Y.L.); sjha@kangwon.ac.kr (S.-J.H.); 2 Department of Biotechnology and Bioengineering, College of ACE, Kangwon National University, Chuncheon 24341, Republic of Korea; 3 Institute of Fermentation of Brewing, Kangwon National University, Chuncheon 24341, Republic of Korea; 4 Department of Environmental Engineering, College of ACE, Kangwon National University, Chuncheon 24341, Republic of Korea; pjaan@kangwon.ac.kr; 5 Department of Biomedical Engineering, College of Engineering, University of North Texas, Denton, TX 76203-5017, USA; youngwook.won@unt.edu

**Keywords:** HSP90 inhibitors, combination therapy, tumor microenvironment, drug resistance, precision oncology

## Abstract

Heat shock protein 90 (HSP90) is a molecular chaperone that plays a pivotal role in the stabilization and functional activation of numerous oncoproteins and signaling molecules essential for cancer cell survival and proliferation. Despite the extensive development and clinical evaluation of HSP90 inhibitors, their therapeutic potential as monotherapies has been limited by suboptimal efficacy, dose-limiting toxicity, and the emergence of drug resistance. Recent studies have demonstrated that combination therapies involving HSP90 inhibitors and other anticancer agents such as chemotherapeutics, targeted therapies, and immune checkpoint inhibitors can enhance anticancer activity, overcome resistance mechanisms, and modulate the tumor microenvironment. These synergistic effects are mediated by the concurrent degradation of client proteins, the disruption of signaling pathways, and the enhancement of antitumor immunity. However, the successful clinical implementation of such combination strategies requires the careful optimization of dosage, administration schedules, toxicity management, and patient selection based on predictive biomarkers. In this review, we provide a comprehensive overview of the mechanistic rationale, preclinical and clinical evidence, and therapeutic challenges associated with HSP90 inhibitor-based combination therapies. We also discuss future directions leveraging emerging technologies including multi-omics profiling, artificial intelligence, and nanoparticle-mediated delivery for the development of personalized and effective combination regimens in oncology.

## 1. Introduction

Heat shock protein 90 (HSP90) is a highly conserved molecular chaperone that regulates the folding, stabilization, and activation of a variety of intracellular proteins, many of which are involved in important processes such as cell proliferation, differentiation, and survival [1,2]. Under physiological conditions, HSP90 functions as a key component in maintaining protein homeostasis and protecting cells from environmental stressors including heat, oxidative damage, and protein misfolding [3]. However, in cancer cells, HSP90 expression is often upregulated, and its functional dependency is greatly enhanced due to the increased burden of mutant or overexpressed oncoproteins [4,5,6].

A wide range of oncogenic signaling molecules, including receptor tyrosine kinases (EGFR, HER2, MET, ALK), intracellular kinases (RAF, MEK, ERK, PI3K, AKT), and transcription factors, are client proteins of HSP90 [7]. Furthermore, HSP90 contributes to the stabilization of tumor suppressor proteins and immune regulators, suggesting a dual role in promoting tumorigenesis and influencing therapeutic resistance [8]. Within the tumor microenvironment, HSP90 activity is further induced by conditions such as hypoxia and nutrient deprivation, promoting cancer cell adaptation, immune evasion, and progression to a more aggressive phenotype [9,10]. HSP90, with its central role in cancer biology, has become an attractive therapeutic target across a variety of tumor types. Over the past two decades, several generations of HSP90 inhibitors have been developed to inhibit the ATPase activity and chaperone function of HSP90. First-generation inhibitors, including geldanamycin and its analog 17-AAG, showed potent in vitro activity, but poor solubility and hepatotoxicity prevented their clinical advancement [11,12,13]. Second-generation synthetic inhibitors such as ganetespib and luminespib have improved pharmacokinetics and selectivity, but still induce a compensatory heat shock response characterized by HSF-1 activation and HSP70/27 upregulation, thereby compromising therapeutic efficacy [14,15,16]. More recently, third-generation inhibitors such as pimitespib, SNX-5422, and XL888 have improved isoform selectivity and reduced toxicity, but their clinical benefit remains minimal, especially in monotherapy [17,18,19,20].

Despite promising preclinical data, HSP90 inhibitor monotherapy has continued to face challenges in clinical translation, including dose-limiting toxicity, immunosuppression, the rapid development of resistance via bypass signaling pathways (e.g., PI3K/AKT, MAPK), alternative receptor activation, and the increased expression of anti-apoptotic chaperones [21,22,23,24,25]. Furthermore, HSP90 inhibition can further limit antitumor immune responses by inducing an immunosuppressive tumor microenvironment [26,27]. These limitations have highlighted the need for strategic combination therapies that can synergistically enhance antitumor efficacy while mitigating toxicity and resistance.

Recent studies have therefore increasingly focused on combining HSP90 inhibitors with chemotherapeutic agents, targeted therapies, and immunotherapies. These combination therapies aim to simultaneously block multiple cancer survival pathways, enhance immune responses, and prevent compensatory mechanisms that impede monotherapy. For example, combination with taxanes or gemcitabine increases DNA damage and cell cycle arrest, combination with targeted therapies (e.g., EGFR, ALK inhibitors) decreases bypass signaling, and combination with immune checkpoint inhibitors (e.g., anti-PD-1/PD-L1) increases T cell activation and tumor immunogenicity [28,29,30,31,32,33,34,35,36]. These multimodal approaches provide a more potent and clinically applicable strategy with which to fully exploit the potential of HSP90 inhibition.

This review examines the current status of HSP90 inhibitors, with a particular focus on combination therapy. We discuss the mechanistic rationale, clinical outcomes, toxicity management, and future directions for optimizing the use of HSP90 inhibitors in modern cancer treatment by leveraging precision oncology and new technologies.

## 2. Classification and Mechanism of HSP90 Inhibitors

### 2.1. Mechanism of Action of HSP90 Inhibitors

**Figure 1:**
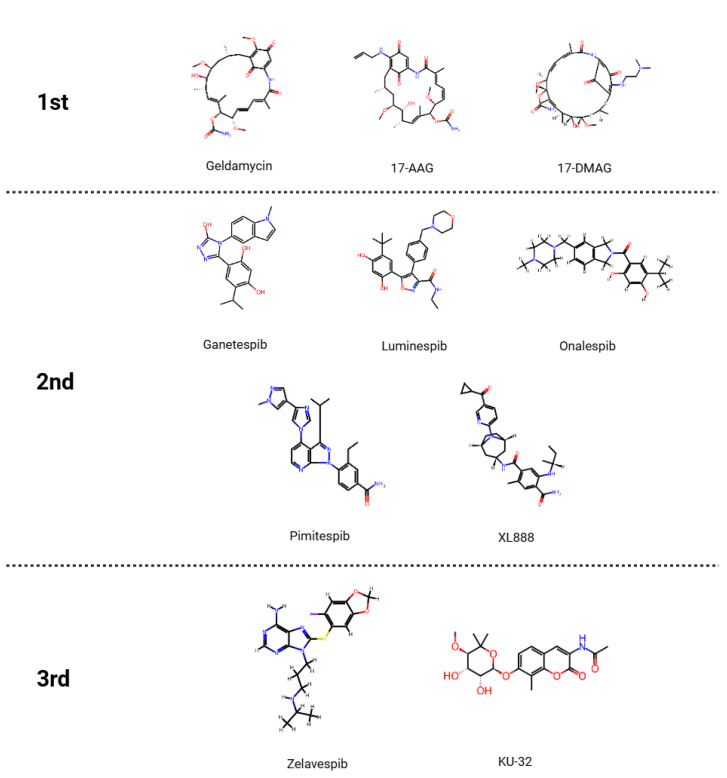
Chemical structures of representative HSP90 inhibitors categorized by generation. 17-AAG: 17-N-allylamino-17-demethoxygeldanamycin; 17-DMAG: 17-dimethylaminoethylamino-17-demethoxygeldanamycin.

First-generation HSP90 inhibitors are represented by natural product-derived geldanamycin and its improved derivative 17-AAG, which directly bind to the N-terminal ATP-binding domain of HSP90 and inhibit its ATPase activity (Figure 1). When ATPase activity is inhibited, the binding between client proteins and HSP90 becomes unstable, and as a result, the client proteins are rapidly degraded via the ubiquitin–proteasome system [12]. However, the problems of low water solubility and high hepatotoxicity have limited their clinical application [13].

Second-generation inhibitors, such as ganetespib, luminespib (AUY922), and onalespib (AT13387), are synthetic small-molecule substances that are improved over first-generation drugs [14] (Figure 1). These inhibitors also act by strongly binding to the HSP90 N-terminal ATP-binding domain and inhibiting its ATPase activity, while offering higher selectivity and improved pharmacokinetic properties [15]. However, these second-generation inhibitors also strongly activate the heat shock response, causing problems such as the increased expression of compensatory chaperone proteins HSP70 and HSP27, limiting their therapeutic efficacy [16].

Third-generation inhibitors are recently developed small-molecule drugs that more selectively target the N-terminal ATP-binding domain of HSP90 and inhibit ATP binding and ATPase activity [17] (Figure 1). In particular, third-generation inhibitors such as pimitespib have higher selectivity for HSP90α and β isoforms than conventional inhibitors, less nonspecific binding to other similar chaperone proteins, and significantly reduced pharmacological toxicity and side effects [19]. Some third-generation inhibitors have been designed as C-terminal domain or HSP90 isoform-specific inhibitors, and have shown clinical efficacy that minimizes the induction of the heat shock response and complements existing limitations [20].

However, even these evolved third-generation inhibitors still have issues to be resolved, such as toxicity problems and the development of treatment resistance, when applied clinically as monotherapy. To overcome this, two mechanistic approaches that can control the function of HSP90 more precisely have recently been attracting attention. First, isoform-selective inhibitors selectively bind to specific isoforms by utilizing the subtle structural differences in the ATP binding pockets between isoforms such as HSP90α, HSP90β, GRP94, and TRAP1 [37,38]. This allows the selective destabilization of oncoproteins by preserving the HSP90 function of non-tumor tissues while inhibiting only the chaperone function through tumor-specific isoforms. For example, TRAP1 inhibitors, a mitochondrial isoform, block energy metabolism in tumor cells by inhibiting oxidative phosphorylation, but do not affect cytosolic HSP90, so they act without inducing the heat shock response [38]. Second, the PROTAC-based inhibition strategy induces ubiquitin-mediated proteasomal degradation by inducing E3 ligase to the target protein through a bifunctional small molecule [39]. This method can induce a more fundamental loss of function than inhibition by directly removing HSP90 itself or the client oncoprotein stabilized by HSP90, rather than simply blocking the enzyme activity [40]. In particular, a chimeric structure linking an HSP90 ligand and an oncogenic client inhibitor can induce selective degradation through the formation of a ternary complex, which is considered a new mechanism of action based on protein fate control [41].

### 2.2. Limitations of HSP90 Inhibitor Monotherapy

#### 2.2.1. Drug Toxicity and Clinical Limitations

HSP90 inhibitors exhibit potent anticancer activity, yet monotherapy approaches have faced limitations in clearly demonstrating clinical therapeutic benefits [1,2]. First-generation inhibitors, such as geldanamycin and 17-AAG, faced restrictions during early-stage clinical trials due to hepatotoxicity and low solubility [42]. These toxicity issues hindered the achievement of sufficient therapeutic concentrations and reduced therapeutic durability [43].

Second-generation inhibitors with improved pharmacological properties (e.g., ganetespib and luminespib) also encountered difficulties in clinical application due to dose restrictions resulting from dose-limiting toxicity (DLT) [44]. Specifically, luminespib has been associated with visual impairments, significantly limiting its long-term administration [45]. Ganetespib has also faced limitations in long-term clinical use due to its gastrointestinal side effects (diarrhea, nausea, etc.) and elevated liver enzymes [46]. Consequently, phase 3 clinical trials reported limited objective response rates (ORRs) and low progression-free survival (PFS), failing to demonstrate sufficient clinical efficacy [21].

Recently developed third-generation inhibitors (e.g., pimitespib, SNX-5422, XL888) have significantly improved toxicity profiles compared to previous generations. However, they still encounter clinical limitations regarding difficulties in achieving therapeutic concentrations and maintaining safety profiles sufficient to demonstrate robust anticancer activity as a monotherapy [9]. Specifically, pimitespib (TAS-116), despite being approved for monotherapy, has shown limited treatment responses in certain cancers, with reported side effects including treatment-related fatigue, elevated liver enzymes, and mild gastrointestinal toxicity in some patient populations [22].

#### 2.2.2. Mechanisms Underlying Resistance Development to HSP90 Inhibitor Monotherapy

One fundamental limitation of HSP90 inhibitor monotherapy is the induction of a heat shock response in cancer cells [23]. HSP90 inhibitors activate heat shock factor-1 (HSF-1), resulting in the upregulation of compensatory chaperone proteins such as HSP70 and HSP27 [24]. These induced proteins inhibit the degradation of client proteins by the ubiquitin–proteasome pathway, thereby preventing apoptosis and enhancing cancer cell survival and therapeutic resistance [25].

**Figure 2:**
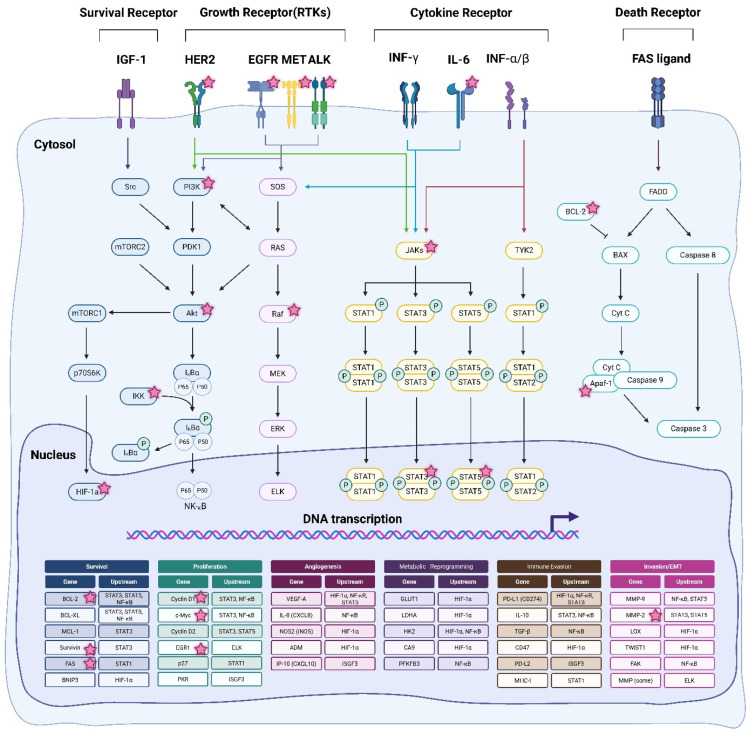
A schematic representation of receptor-mediated oncogenic signaling pathways and transcriptional regulation in cancer. The figure illustrates the major signaling axes activated by survival receptors (e.g., IGF-1R), receptor tyrosine kinases (HER2, EGFR, MET, ALK), cytokine receptors (e.g., IFN-γ, IL-6, IFN-α/β), and death receptors (e.g., FAS ligand). These pathways converge through downstream signaling mediators such as PI3K/Akt, RAS/RAF/MEK/ERK, JAK/STAT, NF-κB, and HIF-1α, regulating the transcription of genes associated with cancer hallmarks including cell survival, proliferation, angiogenesis, metabolic reprogramming, immune evasion, and invasion or epithelial–mesenchymal transition (EMT). The lower panel categorizes representative genes and their upstream regulators involved in each functional hallmark. The pink stars are Client molecules known to be directly inhibited by HSP90. IGF-1R: Insulin-like Growth Factor 1 Receptor; HER2: Human Epidermal Growth Factor Receptor 2; EGFR: epidermal growth factor receptor; MET: Mesenchymal–Epithelial Transition factor (or MET proto-oncogene, receptor tyrosine kinase; ALK: Anaplastic Lymphoma Kinase; INF-γ: Interferon-γ; INF-α/β: Interferon-α/β; IL-6: Interleukin 6; FAS ligand: Fas cell surface death receptor ligand; PI3K: phosphatidylinositol 3-kinase; Akt: Protein Kinase B (PKB), also called Akt, RAS: Rat Sarcoma viral oncogene homolog (small GTPase); Raf: Rapidly Accelerated Fibrosarcoma (serine/threonine-protein kinase); MEK: Mitogen-Activated Protein Kinase Kinase (MAPKK, e.g., MEK1/2 = MAP2K1/2); ERK: Extracellular Signal-Regulated Kinase (MAPK); JAKs: Janus Kinases (JAK1, JAK2, JAK3, TYK2); STAT1, 2, 3, 5: Signal Transducer and Activator of Transcription 1, 2, 3, 5; NF-κB: Nuclear Factor Kappa-Light-Chain-Enhancer of Activated B Cells; HIF 1α: Hypoxia Inducible Factor 1 Alpha [51,52,53,54,55,56,57,58].

HSP90 inhibitor treatment also results in the compensatory activation of the PI3K/AKT and MAPK signaling pathways [47,48] (Figure 2). For instance, following EGFR signaling inhibition, cancer cells often increase the expression and activation of alternative receptors such as MET or IGF-1R, thus activating bypass survival pathways and acquiring therapeutic resistance [49]. The activation of these bypass pathways limits the long-term therapeutic efficacy of HSP90 inhibitor monotherapy, thereby limiting sustained clinical efficacy due to the rapid adaptation of cancer cells [50].

Additionally, HSP90 inhibitors may promote immune evasion and create an immunosuppressive tumor microenvironment by enhancing the functional activity and accumulation of myeloid-derived suppressor cells (MDSCs) and regulatory T cells (Tregs) [26]. Consequently, HSP90 inhibitor monotherapy alone may fail to effectively stimulate antitumor immune responses, reducing therapeutic efficacy without combination with immunotherapy [27].

In summary, these toxicity issues, clinical efficacy limitations, and resistance mechanisms highlight the difficulty of using HSP90 inhibitors as monotherapy, emphasizing the necessity of strategic therapeutic combinations for more effective and safe clinical applications.

### 2.3. Necessity and Clinical Significance of Combination Therapy

To address the limited efficacy and rapid development of resistance associated with HSP90 inhibitor monotherapy, combination strategies involving diverse anticancer agents have received significant attention in recent years [59,60]. Combination therapy offers a strategic approach by utilizing agents with distinct mechanisms of action to maximize therapeutic efficacy, reduce drug dosages, and prevent the emergence of resistance. Among these, HSP90 inhibitor-based combinations are particularly promising due to the central role of HSP90 in stabilizing multiple oncogenic proteins and signaling pathways [61,62].

HSP90 inhibitors exert synergistic effects with chemotherapeutic and targeted agents primarily through the degradation of intracellular client proteins. In chemotherapy, they enhance cytotoxicity by promoting the degradation of proteins involved in cell cycle regulation and DNA repair, thereby sensitizing tumor cells to apoptosis and overcoming drug resistance [28,63,64]. For example, the third-generation HSP90 inhibitor pimitespib has shown enhanced antitumor activity in pancreatic and ovarian cancer models when combined with gemcitabine or cisplatin [29,30]. Similarly, in targeted therapy settings, HSP90 inhibitors have been shown to suppress the expression of resistance-associated proteins and delay resistance development. Clinical trials have demonstrated that combining luminespib with ALK inhibitors in ALK inhibitor-resistant non-small cell lung cancer (NSCLC) improved the clinical response, and that HSP90 inhibitors also restored sensitivity to BRAF/MEK inhibitors in resistant melanoma models [31,32,33].

In the field of immunotherapy, HSP90 inhibitors have emerged as promising agents that modulate the immunosuppressive tumor microenvironment. They can downregulate PD-L1 expression in tumor cells, reduce the activity of immunosuppressive regulatory T cells (Tregs) and myeloid-derived suppressor cells (MDSCs), and enhance the efficacy of immune checkpoint inhibitors such as anti–PD-1/PD-L1 antibodies [34]. Preclinical and early clinical data support this synergy; for instance, the combination of pimitespib and nivolumab showed improved efficacy over monotherapy, while the co-administration of XL888 and pembrolizumab induced immune activation and antitumor effects in treatment-resistant pancreatic cancer models [35,36].

As these combination approaches continue to show clinical promise, the need for precision medicine strategies to guide optimal drug selection, dosage, and scheduling is increasingly evident. Future advances will likely involve the integration of molecular profiling, biomarker-based patient stratification, and computational modeling to refine HSP90-based combination therapies. In the following sections, we explore the detailed molecular mechanisms and clinical outcomes of combination therapies involving HSP90 inhibitors with chemotherapeutic, targeted, and immunotherapeutic agents (Table 1).

## 3. Mechanisms and Clinical Rationale for HSP90 Inhibitor Combination Therapy

### 3.1. Chemotherapeutic Agents

#### 3.1.1. Taxane

The taxane drugs paclitaxel and docetaxel inhibit microtubule depolymerization and promote their dynamic stabilization, leading to cell division arrest at the G2/M phase and inducing apoptosis [65]. Combining HSP90 inhibitors with taxane drugs exhibits strong synergistic effects by simultaneously targeting microtubule dynamics, cell cycle regulation, and apoptosis pathways [66].

HSP90 inhibitors enhance cell cycle arrest by reducing the stability of G2/M regulatory proteins such as cyclin B1, CDK1, and CDC25C, promoting their degradation via the ubiquitin–proteasome pathway [67]. Additionally, the inactivation of major survival signaling pathways such as AKT, ERK, and RAF by HSP90 inhibitors increases apoptosis induced by taxane-based drugs [68].

In clinical trials involving patients with trastuzumab-resistant HER2-positive metastatic breast cancer, the combination of ganetespib (150 mg/m^2^ on days 1, 8, and 15), paclitaxel (80 mg/m^2^ on days 1, 8, 15, and 22), and trastuzumab (2 mg/kg weekly) demonstrated acceptable safety without DLT in nine patients, and a recommended phase 2 dose (RP2D) of ganetespib (150 mg/m^2^) was established [44]. Furthermore, in patients with high-risk HER2-negative early breast cancer (neoadjuvant setting, I-SPY2 trial), combining ganetespib (150 mg/m^2^ every 3 weeks) with paclitaxel (weekly for 12 weeks) improved pathological complete response (pCR) rates to 26–38% compared to 22% in the control group; however, it did not meet the criteria for progression to phase III clinical trials, and it remains uncertain whether this combination substantially improves outcomes compared to standard adjuvant therapy [69].

In the GALAXY-2 phase III clinical trial involving 672 patients with advanced lung adenocarcinoma, the combination of ganetespib (150 mg/m^2^ on days 1 and 15) and docetaxel (75 mg/m^2^, 21-day cycle) showed a modest improvement in overall survival compared with docetaxel alone. However, there was no significant difference in progression-free survival (PFS: 4.2 vs. 4.3 months, HR 1.16, P = 0.119), and grade 3/4 neutropenia was observed in 30.9% of patients in the combination group versus 25% in the monotherapy group, indicating that the combination had limited additional benefits [70].

In a clinical study involving patients with progressive triple-negative breast cancer (TNBC), the combination of onalespib (260 mg/m^2^) and paclitaxel (80 mg/m^2^) resulted in an overall response rate (ORR) of 20%, including three complete responses, with a median PFS of 2.9 months [71].

Several HSP90 inhibitors have shown efficacy in preclinical and early clinical trials, but have failed to show significant clinical effects in late-stage clinical trials. In the GALAXY-2 phase 3 study in patients with advanced non-small cell lung cancer, the combination of ganetespib and docetaxel did not improve overall survival or progression-free survival compared to docetaxel alone [70]. This result may be related to the lack of patient selection based on HSP90 client protein dependency. HSP90 regulates various oncoproteins, but not all tumors are dependent on it. If treatment is applied without molecular criteria, the therapeutic effect may be limited in patient groups with various characteristics. A retrospective analysis showed that patients with HER2 amplification, ALK rearrangement, and mutant EGFR may respond better to HSP90 inhibition [72]. These results suggest the need for biomarker-based patient selection in future clinical trials.

#### 3.1.2. Gemcitabine

Gemcitabine is a structural analogue of deoxycytidine (dC) that disrupts DNA replication in cancer cells and induces apoptosis [73]. After entering the cell, gemcitabine undergoes sequential phosphorylation steps to become the active metabolite gemcitabine triphosphate (dFdCTP) [74]. The active gemcitabine is incorporated into the DNA strand during DNA synthesis, preventing DNA polymerase activity and terminating DNA synthesis by inhibiting chain elongation. Gemcitabine-induced DNA damage activates survival signaling pathways that trigger the DNA damage response (DDR) in cancer cells, facilitating DNA damage repair processes [75].

In this process, HSP90 inhibitors enhance the anticancer effect by increasing the sensitivity of cancer cells to gemcitabine treatment, while simultaneously suppressing compensatory survival signals [76,77]. Gemcitabine can reduce cytotoxicity by activating the DNA damage response and survival pathways such as AKT and ERK, while HSP90 inhibitors reduce resistance and induce apoptosis by impairing the stability of these key proteins [74,75,76,77,78,79,80,81].

Clinical studies evaluating the combination of tanespimycin (17-AAG) and gemcitabine in patients with advanced pancreatic cancer have reported modest clinical responses, with objective response rates (ORR) of around 10–15%, disease control rates (DCR) of 50–60%, a median PFS of 4.2 months, and a median overall survival (OS) of 7.1 months [82]. Additionally, in patients with advanced ovarian and peritoneal cancer, the combination of tanespimycin (154 mg/m^2^) and gemcitabine (750 mg/m^2^) demonstrated preliminary efficacy, showing a partial response (PR) rate of 8.3% (1/12) and stable disease (SD) rate of 50%; however, the extent of client protein inhibition was limited [83].

Additionally, the experimental HSP90 inhibitors ICPD47 and ICPD62 demonstrated strong synergistic effects in combination with gemcitabine and 5-FU in pancreatic cancer cell lines, with combination index (CI) values of 0.16–0.33, and showed a 2.4-fold reduction in EC_50_ under hyperthermia conditions [84].

These results demonstrate that the combination strategy of HSP90 inhibitors and gemcitabine is unlikely to lead to clinical outcomes based solely on expectations of mechanistic synergy. In fact, in clinical settings, the expected molecular interactions have not consistently translated into therapeutic effects due to the induction of a heat shock response by HSP90 inhibition, heterogeneity in the range of client protein inhibition, and inhomogeneity in drug distribution within tumors. In particular, the myelotoxicity and hepatotoxicity of gemcitabine overlapped with the intrinsic toxicity of HSP90 inhibitors, resulting in a structural bottleneck that limited the therapeutic window. Despite the strong synergy and potential to overcome resistance in preclinical settings, the failure to reproduce these effects in clinical settings suggests that combination strategies cannot be optimized solely by the mechanistic complementarity of drug combinations, and that precise combination design that comprehensively reflects the tumor biology context, drug accumulation kinetics, and patient-specific resistance characteristics is essential.

#### 3.1.3. Cisplatin

Cisplatin is a representative platinum-based anticancer drug that induces the DNA cross-linking and apoptosis of cancer cells, but its therapeutic efficacy is limited due to the development of resistance after repeated administration [85,86]. To overcome this limitation, combination therapy with HSP90 inhibitors has been actively studied in preclinical studies. HSP90 inhibitors have been reported to inhibit the stability of key proteins involved in DNA damage response, thereby inhibiting the DNA repair process and enhancing the cisplatin-induced apoptosis effect. In fact, it has been shown that when used in combination, DNA damage accumulation, cell cycle arrest, and apoptosis are effectively induced [85,86,87,88].

For instance, in a diffuse large B-cell lymphoma (DLBCL) model, the combined administration of 17-AAG and cisplatin increased apoptosis rates more than two-fold compared to cisplatin alone and further enhanced tumor suppression by approximately 50–70% in vivo, suggesting an ability to overcome cisplatin resistance [89]. In ovarian cancer, the combination of onalespib and cisplatin demonstrated synergistic effects, reducing the tumor volume by approximately 70% compared to cisplatin alone, extending survival from an average of 30 days to up to 50 days, and decreasing cell viability by an additional 20–30% [90]. In a nasopharyngeal carcinoma (NPC) model, combining AUY922 and cisplatin significantly increased apoptosis compared to cisplatin alone (*p* < 0.05), markedly inhibited tumor growth in xenograft mice (*p* < 0.05), and overcame drug resistance without notable toxicity [91].

These results suggest that the combination of HSP90 inhibitors and cisplatin may overcome the resistance of existing chemotherapy and enhance the therapeutic efficacy in various cancers. However, despite these preclinical results, the combination therapy has not entered the clinical stage. This is due to a combination of problems, such as the expected increased toxicity of combination therapy, such as hepatotoxicity and nephrotoxicity, the lack of safety and pharmacokinetic data in the early clinical stage, the limited efficacy and unfavorable pharmacokinetic characteristics of some HSP90 inhibitors, and the discontinuation of commercial development [92]. In particular, the absence of a biomarker-based patient selection strategy makes it difficult to define specific tumor subtypes that can increase the clinical applicability of the combination therapy [93].

### 3.2. Targeted Therapeutic Agents

#### 3.2.1. Membrane Receptor-Targeted Inhibitors

HER2 and EGFR inhibitors either directly bind to receptor tyrosine kinases (RTKs) on the cell membrane, competitively blocking the ATP-binding site, or bind to the extracellular domain to prevent receptor dimerization [94,95]. EGFR inhibitors (e.g., erlotinib, gefitinib) inhibit ATP binding to the tyrosine kinase domain of EGFR, preventing receptor autophosphorylation, thereby inhibiting downstream signaling through the RAS-RAF-MEK-ERK and PI3K-AKT-mTOR pathways in cancer cells [96]. Similarly, HER2 inhibitors (e.g., trastuzumab, lapatinib) bind to the extracellular or intracellular domains of HER2, disrupting HER2 activation and dimerization and inhibiting the same downstream signaling pathways mediated by HER2 [31]. However, signal blockade by these HER2 and EGFR inhibitors induces compensatory responses in cancer cells, such as the overexpression and dimerization of other RTKs (e.g., HER3, MET), activating bypass pathways and ultimately leading to therapeutic resistance [97].

HSP90 inhibitors can fundamentally inhibit the stability and expression of RTK receptors, including HER2 and EGFR, thereby blocking the bypass RTK activation induced by the administration of targeted inhibitors alone. Therefore, the combined use of the two drugs acts as a dual blocking strategy that inhibits not only the activity but also the expression of RTKs, and can effectively overcome treatment resistance and enhance the anticancer effect [98,99,100].

In a clinical study of 31 patients with trastuzumab-resistant HER2-positive breast cancer who had previously progressed on trastuzumab therapy, the combination of tanespimycin (450 mg/m^2^) and trastuzumab demonstrated significant anticancer activity, with an ORR of 22%, clinical benefit rate (CBR) of 59%, median PFS of 6 months, and median overall survival (OS) of 17 months [101]. Additionally, a triple combination of ganetespib (150 mg/m^2^), paclitaxel (80 mg/m^2^), and trastuzumab (2 mg/kg) in nine patients with trastuzumab-resistant HER2-positive metastatic breast cancer showed a PR of 22% and SD of 52%, with no DLT and good safety [44].

However, in patients with EGFR-mutated non-small cell lung cancer (NSCLC), the combination of AUY922 (70 mg/m^2^ once weekly) and erlotinib showed modest efficacy (PR 16%), but the clinical benefit was limited due to an increase in adverse effects, such as night blindness [102].

A combined therapy comprising HER2 and EGFR inhibitors and HSP90 inhibitors showed improved therapeutic efficacy and the possibility of overcoming resistance in some cancers, but showed a limited effect in EGFR-mutated non-small cell lung cancer (NSCLC). This is interpreted to be the result of the existence of complex resistance mechanisms such as T790M secondary mutation and MET amplification in EGFR-mutated NSCLC, and the inability of HSP90 inhibitors to secure pharmacodynamic characteristics sufficient to stably degrade EGFR protein. Therefore, the effect of combination therapy may vary depending on the molecular characteristics of the cancer and the pharmacological characteristics of the combination drugs, and the establishment of a precise clinical strategy that reflects this is required.

#### 3.2.2. Intracellular Signaling Pathway Inhibitors

Intracellular signaling pathways such as ALK, BRAF/MEK, and PI3K/AKT/mTOR are essential for the growth and survival of cancer cells. Unlike EGFR and HER2 inhibitors that target receptor tyrosine kinases located on the cell surface, ALK, BRAF/MEK, and PI3K/AKT/mTOR inhibitors act on non-receptor kinases in intracellular signaling pathways critical for cancer cell proliferation and survival [103,104]. These signaling molecules are client proteins of HSP90, and their stability and activation depend on the chaperone function of HSP90 [2]. HSP90 inhibitors disrupt the structural stability of these signaling proteins, promoting their degradation via the ubiquitin–proteasome pathway, thereby inhibiting signaling and suppressing cancer cell growth [1].

Anaplastic lymphoma kinase (ALK) is activated predominantly as an EML4-ALK fusion protein, especially in non-small cell lung cancer (NSCLC), and promotes cancer cell proliferation [105]. HSP90 inhibitors reduce the stability of ALK fusion proteins and promote their degradation, enhancing therapeutic sensitivity to ALK inhibitors [106].

BRAF and MEK proteins activate the MAPK pathway, driving proliferation and survival, particularly in cancers harboring BRAF V600E mutations, such as melanoma [107]. HSP90 inhibitors destabilize BRAF and MEK proteins, inhibit MAPK signaling, and enhance the efficacy of BRAF/MEK inhibitors [108].

The PI3K/AKT/mTOR pathway is activated in many carcinomas, driving cell growth and therapeutic resistance. HSP90 inhibitors impair the structural stability of PI3K, AKT, and mTOR proteins, suppressing pathway activation and survival signaling. When combined with PI3K/AKT/mTOR inhibitors, HSP90 inhibitors more effectively reduce the expression and activation of pathway components, improving therapeutic efficacy and overcoming drug resistance compared to monotherapy [109].

In ALK-positive NSCLC patients, preclinical studies showed that the combination of AT13387 and crizotinib resulted in superior tumor inhibition and delayed resistance compared to crizotinib monotherapy. Early clinical trials established the maximum tolerated dose (MTD) and recommended RP2D without significant safety issues, and further evaluations are currently ongoing in phase 2 clinical trials [110]. Additionally, in patients with BRAF V600-mutant melanoma, the triple combination of vemurafenib, cobimetinib, and XL888 demonstrated significant antitumor activity, including an ORR of 76%, median PFS of 7.6 months, and OS of 41.7 months; however, frequent dose adjustments were required [111].

On the other hand, a combined therapy comprising PI3K/AKT/mTOR inhibitors and HSP90 inhibitors has been reported to induce anticancer synergy through complementary mechanisms in preclinical studies, but studies in actual clinical practice are limited due to concerns about increased toxicity, limitations in the therapeutic index, and a lack of evidence on optimal combination strategies. In particular, there is a lack of reliable biomarkers that can predict the response to combination therapy, which makes patient selection and the evaluation of treatment response difficult, and this is thought to be one of the major factors lowering the possibility of clinical application [112,113].

Conversely, clinical data on combining PI3K/AKT/mTOR inhibitors with HSP90 inhibitors remain limited, necessitating further studies to assess clinical efficacy and safety. Therefore, for patients with ALK-positive NSCLC and BRAF V600-mutant melanoma, HSP90 inhibitor-based combination therapy is a promising strategy to overcome resistance and enhance therapeutic outcomes. However, rigorous clinical evaluation and optimization strategies for combinations targeting the PI3K/AKT/mTOR pathway are essential for future progress.

### 3.3. Immunotherapeutic Agents

#### 3.3.1. PD-1 and PD-L1 Inhibitors

PD-1 (Programmed cell death protein-1) is an inhibitory immune checkpoint receptor expressed on the surface of activated T cells, and PD-L1 (Programmed death-ligand 1) is a ligand expressed mainly on tumor cells [114]. When PD-L1 on cancer cells binds to PD-1 on T cells, it inhibits signaling via the PI3K/AKT and Ras/MEK/ERK pathways downstream of the T cell receptor (TCR) through SHP-1/SHP-2 phosphatases [115,116]. This interaction is the primary mechanism by which cancer cells promote immune evasion by significantly reducing T cell proliferation, cytotoxicity, and cytokine secretion [117].

PD-1 inhibitors (nivolumab, pembrolizumab) specifically bind to the PD-1 receptor on T cells, block the interaction of PD-L1 and PD-L2 ligands on tumor cells with PD-1, and restore suppressed T cell function to activate anticancer immune responses [118]. PD-L1 inhibitors (atezolizumab, durvalumab) selectively bind to PD-L1 on tumor cells, reverse T cell inhibition by directly blocking PD-L1 binding to PD-1, and enhance T cell-mediated immune responses [119]. HSP90 inhibitors inhibit the chaperone function of HSP90 required for the stabilization and expression of the PD-L1 protein on cancer cell surfaces, significantly reducing PD-L1 expression in tumors [120]. Thus, combined use with HSP90 inhibitors can further enhance the therapeutic effects of PD-1 and PD-L1 inhibitors [121]. For instance, the combination of pimitespib and PD-1 inhibitors significantly reduced FOXP3-expressing activated Treg cells and improved CD8^+^ T cell activity through STAT5 degradation in a gastric cancer model, demonstrating significant tumor-suppressive effects compared to monotherapy [122]. Furthermore, the combination of TAS-116 and nivolumab demonstrated an ORR of 16%, inhibited Treg cells, enhanced immune activation, and suggested potential for partially improving the efficacy of PD-1 immunotherapy in patients with MSS colorectal cancer, with a tolerable toxicity profile and no DLT [123]. Additionally, combining HSP90-targeting photodynamic therapy (HS201-PDT) with an anti-PD-L1 antibody significantly increased tumor suppression and survival rates compared to monotherapy (*p* < 0.01), increased the activation of CD8^+^ T cells, and exhibited an abscopal effect in distant tumors through immune activation mediated by the CXCR3-related pathway [124].

These results are mostly based on limited preclinical models or small data observed in early clinical trials, and it cannot be ruled out that the immune modulatory mechanisms of combination therapy may work differently depending on the specific tumor type. Therefore, there is not yet sufficient evidence to confirm whether HSP90 inhibitors can consistently enhance the therapeutic efficacy of immune checkpoint inhibitors, and follow-up studies including clinical efficacy and the validation of predictive biomarkers are essential.

#### 3.3.2. CTLA-4 Inhibitors

CTLA-4 (cytotoxic T-lymphocyte-associated protein 4) is an immune checkpoint receptor expressed on early activated T cells, competitively binding the B7 ligand (CD80/CD86) on antigen-presenting cells (APCs) against the T cell activation receptor CD28 [114]. CTLA-4 exhibits higher affinity for B7 ligands than CD28, suppressing T cell activation and limiting anti-cancer immune responses [125]. CTLA-4 inhibitors (ipilimumab, tremelimumab) block CTLA-4 binding, thus promoting CD28-B7 interactions and amplifying T cell activation and tumor-specific cytotoxic responses [118,126].

Preclinical studies have reported augmented anti-cancer immune effects when CTLA-4 inhibitors are combined with HSP90 inhibitors. An important mechanism underlying this combination is the modulation of the tumor microenvironment by HSP90 inhibitors, as demonstrated by reduced PD-L1 expression on tumor cell surfaces and the suppression of regulatory T cell (Treg) activity and function [22,50,127]. This effect enhances the T cell activation induced by CTLA-4 inhibitors, aiding in overcoming the immunosuppressive tumor microenvironment [128].

In preclinical melanoma models, the combination of Ganetespib and a CTLA-4 inhibitor has shown anticancer effects in melanoma and colon cancer models through a decrease in immunosuppressive regulatory T cells and an increase in the antitumor activity of CD8+ T cells [120]. However, to date, clinical trials on the combination of a CTLA-4 inhibitor and an HSP90 inhibitor have rarely been reported. This is thought to be due to the risk of unpredictable immune-related adverse reactions due to the broad effects of both drug classes on the immune system, and the lack of established clinical standards for the optimal dose and timing of administration for combination therapy. The immunomodulatory and antitumor effects observed in the preclinical stage suggest the potential of the combination strategy [120], but more sophisticated clinical approaches, such as the quantitative assessment of immunotoxicity, screening for tumor subtypes suitable for combination therapy, and the discovery of biomarkers predicting response, are needed to clinically scale this up.

## 4. Toxicity Management, Optimization Strategies, and Future Research Directions for HSP90 Inhibitor Combination Therapy

### 4.1. How to Overcome Toxicity Management and Resistance When Using HSP90 Inhibitor Combination Therapy

Combination therapy using HSP90 inhibitors is a well-known strategy that can enhance synergistic antitumor effects and improve therapeutic efficacy by simultaneously targeting multiple targets, and can also reduce toxicity by reducing the dose of each drug and delaying the development of resistance, which is common in monotherapy [129,130]. For example, the co-administration of HSP90 inhibitors with different mechanisms of action and other anticancer drugs at low doses can minimize side effects and produce similar therapeutic effects compared to using a single drug at high doses, thereby improving patients’ treatment tolerance and quality of life [130]. On the other hand, systematic toxicity management is essential because there is a possibility that unexpected side effects may increase due to drug interactions and overlapping toxicities resulting from combination therapy [131]. In fact, it has been reported that when HSP90 inhibitors and immunotherapy or chemotherapy were co-administered, the risk of side effects of all grades increased compared to monotherapy, and the incidence of severe side effects also significantly increased [132]. In combination chemotherapy, when ganetespib or onalespib was used with paclitaxel, platinum, etc., adverse effects such as diarrhea, leukopenia, and peripheral neuropathy frequently occurred, and in such cases, predefined dose reductions were applied to approximately 17% of patients or toxicity was managed with symptomatic treatment [69,133]. In combination with immune checkpoint inhibitors, criteria such as the concomitant use of steroids, the administration of immunosuppressants, and the exclusion of patients with underlying autoimmune diseases were applied, considering the occurrence of autoimmune adverse reactions (irAEs), and in preclinical trials, ganetespib showed an immunomodulatory effect that alleviated anti-PD-1 antibody hypersensitivity and increased T cell activity [60,134]. Therefore, when implementing HSP90 inhibitor combination therapy, an active toxicity management strategy is required, such as closely monitoring patients’ adverse reactions, adjusting the dosing schedule as necessary, or selecting a combination of drugs with non-overlapping toxicity profiles.

HSP90 inhibitor combination therapy has also been highlighted as an effective approach for overcoming resistance resulting from tumor cell heterogeneity and the activation of multiple signaling pathways. The simultaneous use of HSP90 inhibitors in combination with drugs having different mechanisms of action can simultaneously target various clones within a cancer cell population and delay or suppress the development of adaptive drug resistance, which typically emerges over time with single-drug treatments. This approach simultaneously blocks multiple pathways essential for cancer cell survival, thereby hindering cancer cell survival via alternative bypass pathways [131]. Furthermore, even if resistance to targeted therapeutic agents has already developed, it has been reported that the vulnerability of resistant cancer cells can be exploited and therapeutic responses can be restored; this is by further combining agents with novel mechanisms of action, such as HSP90 inhibitors [48]. Ultimately, to effectively overcome drug resistance through combination strategies, it is essential to thoroughly understand the resistance mechanisms of tumors and select drugs capable of specifically targeting these mechanisms.

### 4.2. Optimization of HSP90 Inhibitor Combination Therapy

To maximize the effectiveness of combination therapy using HSP90 inhibitors, it is essential to move beyond empirically selected or non-optimized drug pairings and instead systematically design combination regimens that incorporate rational drug selection, dosage adjustments, administration sequence, and scheduling. In this context, various quantitative models and indices have been developed to evaluate the synergistic effects of drug combinations, ranging from classical in vitro approaches such as the Chou–Talalay method to more complex computational modeling techniques [135,136]. Despite these tools, accurately predicting which combinations will yield optimal therapeutic outcomes remains challenging, and effective regimens have often been identified through trial-and-error experimentation or clinical intuition. Moreover, pharmacokinetic and pharmacodynamic interactions between drugs can lead to suboptimal efficacy or unexpected toxicities, underscoring the need for sophisticated optimization strategies. Therefore, during the development of HSP90 inhibitor-based combinations, a thorough analysis of each agent’s pharmacological profile and interaction potential is critical, along with careful consideration of the administration timing, sequence, and dose to minimize adverse effects and maximize clinical benefit [132,137].

Recently, in silico assay approaches have attracted attention for their potential to more accurately predict the pharmacological properties of HSP90 inhibitors and develop effective combination therapies. HSP90 inhibitors are molecular chaperones that play an important role in stabilizing various tumor signaling proteins, and their binding affinity and inhibitory activity directly affect therapeutic efficacy [3]. Recent studies have utilized advanced computer-based analytical techniques, such as molecular docking and molecular dynamics (MD) simulation, to accurately predict binding sites between HSP90 inhibitors and target proteins and clearly identify the structure–activity relationships of the drugs [138,139,140]. Notably, during the discovery of novel marine-derived HSP90 inhibitors, comprehensive in silico analyses, including ADMET (absorption, distribution, metabolism, excretion, and toxicity) predictions, contributed to selecting candidate substances with high drug efficacy and low toxicity [141].

Furthermore, when two or more drugs are administered in combination for various cancer treatments, accurately understanding the pharmacological properties and pharmacokinetic interactions of each drug is central to formulating effective combination strategies. In silico analyses based on machine learning and artificial intelligence (AI) have shown excellent results in quantitatively evaluating the synergistic or antagonistic effects between drugs and predicting optimal combination doses and administration schedules [142,143,144,145]. Based on these technological advancements, combination therapy using HSP90 inhibitors with other anticancer drugs is expected to be optimized through computer modeling and machine learning analyses. This approach will become essential for quickly evaluating the efficacy and toxicity of combination therapies at pre-clinical and clinical trial stages and establishing more precise personalized treatment strategies.

### 4.3. Biomarker Limitations and Opportunities for Precise Application of HSP90 Inhibitor-Based Combination Therapy

Recent studies have pointed out the lack of reliable biomarkers across various cancer types as a major obstacle to the precise application of HSP90 inhibitor-based combination therapy. Due to tumor heterogeneity, the discovery and validation of biomarkers is very difficult, and even within the same cancer type, each tumor responds differently to HSP90 inhibition, making it difficult to find a universally applicable biomarker [146]. As a result, biomarkers that can predict the treatment response are still rare, and the drug response is influenced by various factors and the tumor characteristics of each patient vary, making it difficult to identify such predictive markers. Traditionally, the effect of HSP90 inhibitors is confirmed by using the increase in HSP70 induction or the degree of client protein degradation as pharmacodynamic indicators, but the changes in these indicators often do not match the clinical effect [147]. This is reported to be because not all tumors follow the same client protein degradation pathway following the expected inhibitor binding, adding to the difficulty in identifying reliable pharmacodynamics/response biomarkers [146]. Nevertheless, recent studies have suggested the possibility of discovering new biomarkers. For example, approaches based on multi-gene/protein signatures are being explored, and a machine learning study predicted sensitivity to an HSP90 inhibitor (17-AAG) with an accuracy of approximately 92% using a panel of 16 proteins [147]. In addition, the expression level of HSP90 in tumor tissue itself is being reexamined as a biomarker. In patients with advanced colorectal cancer, the group with a high expression of HSP90α/β in the tumor had a significantly worse prognosis, but paradoxically, the treatment response to the HSP90 inhibitor pimitesrib was better, showing an enhanced effect especially when administered in combination with chemotherapy or targeted therapy [148]. In this way, the context according to molecular subtype is also highlighted as an important factor. Tumors addicted to specific oncoproteins, such as EGFR mutations or ALK translocations, are particularly sensitive to HSP90 inhibition [149], and proteins such as ADI1 and RRP1 have been suggested as indicators confirming HSP90 inhibition through proteomic studies in lung adenocarcinoma models with EGFR/ALK mutations; in addition, proteins such as ASS1, ITCH, and UBE2L3 were suggested to be response predictors for each molecular subtype [150]. Furthermore, biomarker clues are being discovered in terms of the tumor microenvironment (TME). It was reported that HSP90 inhibitors can regulate the immune microenvironment, and it was confirmed in an animal model that when pimitespib was used, immunosuppressive regulatory T cells (FOXP3 high-expressing Tregs) were selectively reduced and the activation of antigen-specific CD8+ T cells was enhanced, showing a synergistic effect when used in combination with a PD-1 immune checkpoint inhibitor [151]. These recent research trends suggest that integrating biomarkers such as HSP90 client protein profiles, TME immune signatures, and molecular subtypes may enable a precision medicine approach to improving patient stratification and selection in future HSP90 inhibitor combination therapy trials, thereby identifying the patient populations most likely to benefit from such therapy [152].

### 4.4. Future Research Direction and Clinical Applicability

In future research on HSP90 inhibitor combination therapies, precision medicine approaches based on the molecular and biological characteristics of cancer are expected to be further strengthened. Advanced technologies are being developed to predict the most suitable drug combinations for each patient by integrating and analyzing multiple omics data, such as genomic, proteomic, and transcriptomic data, from each patient’s tumor, using artificial intelligence (AI)-based models [137,153]. Additionally, research is expanding to incorporate clinical data and real-world evidence from patient treatments to more accurately predict the efficacy and toxicity of combination therapies [154].

Clinically, opportunities for applying various innovative HSP90 inhibitor combination therapies are also increasing. Combination therapy using HSP90 inhibitors has already demonstrated improved treatment outcomes and effectiveness in several carcinoma types and is anticipated to become an essential component of cancer treatment strategies [155]. Furthermore, it is expected that these multi-drug combination therapies will be adopted as standard treatments based on accumulated clinical evidence, ushering in an era of truly personalized medicine, wherein optimal combination therapies are selected based on each patient’s unique biomarker profile.

## 5. Conclusions

Once limited to monotherapy, HSP90 inhibitors are now re-emerging as an important component of combination therapies in oncology. When combined with anticancer agents, targeted therapies, or immune checkpoint inhibitors, HSP90 inhibitors exert powerful synergistic effects by modulating patient tumor protein degradation and resistance-related pathways. Combination therapies not only enhance efficacy but also offer the opportunity to reduce toxicity through dose adjustments. However, the clinical application of these combination therapies requires the careful consideration of pharmacodynamic interactions, overlapping toxicities, and a personalized treatment design based on biomarker stratification. In the future, the integration of computational modeling, artificial intelligence, and multi-omics analyses will play a key role in identifying optimal drug partners and formulating dosing strategies. In addition, innovative platforms such as nanoparticle-based delivery systems and isoform-selective HSP90 inhibitors are expected to further improve the safety and efficacy of these therapies. Ultimately, well-designed clinical trials incorporating these advances are essential to realize the full therapeutic potential of HSP90 inhibitor-based combination therapies and establish them as a standard approach to precision cancer treatment.

## Figures and Tables

**Table 1:** Combination strategies of HSP90 inhibitors by generation and clinical development status.

Generation	Combination Therapy	Hsp90 Inhibitors	Types of Cancer	Preclinical and Clinical	NCT Number
I	Gemcitabine	Tanespimycin (17-AAG)	Metastatic pancreatic cancer (Stage IV)	Phase II	NCT00368590
I	Gemcitabine	Tanespimycin (17-AAG)	Ovarian and peritoneal cancer	Phase II	NCT00387722
I	Cisplatin	Tanespimycin (17-AAG)	Diffuse large B-cell lymphoma (DLBCL)	Preclinical	
I	Trastuzumab	Tanespimycin (17-AAG)	HER2-positive metastatic breast cancer	Phase II	NCT00027846
II	Paclitaxel + Trastuzumab	Ganetespib	HER2-positive metastatic breast cancer	Phase I	NCT01273473
II	Paclitaxel	Ganetespib	HER2-negative early-stage breast cancer	Phase II	NCT01042379
II	Paclitaxel	Onalespib	Advanced triple-negative breast cancer (TNBC)	Phase I	NCT02474173
II	Docetaxel	Ganetespib	Advanced non-small cell lung cancer (NSCLC)	Phase III	NCT01798485
II	Gemcitabine	ICPD47 or ICPD62	Pancreatic cancer cell lines (MIA PaCa-2, PANC-1)	Preclinical	
II	Cisplatin	Luminespib (AUY922)	Nasopharyngeal carcinoma (NPC)	Preclinical	
II	Cisplatin	Onalespib	Ovarian and peritoneal cancer	Preclinical	
II	Erlotinib	Luminespib (AUY922)	EGFR-mutant non-small cell lung cancer (NSCLC)	Phase I	NCT01259089
II	paclitaxel + Trastuzumab	Ganetespib	HER2-positive metastatic breast cancer	Phase I	NCT00096391
II	Crizotinib	Onalespib	ALK-positive non-small cell lung cancer (NSCLC)	Preclinical	
II	Crizotinib	Onalespib	ALK-positive non-small cell lung cancer (NSCLC)	Phase I/II	NCT01712217
II	Vemurafenib (BRAF inhibitor) + Cobimetinib (MEK inhibitor)	XL888	BRAF V600E-mutant melanoma	Phase I	NCT01657591
II	Atezolizumab (PD-L1 inhibitor)	Pimitespib	Metastatic solid tumors	Preclinical	
II	Nivolumab (PD-1 inhibitor)	Pimitespib	Advanced solid tumors	Phase I	NCT02872116
II	Clone 9H10 (CTLA-4 inhibitor)	Ganetespib	Melanoma	Preclinical	

## Data Availability

The datasets presented in this article are not readily available because the data are part of an ongoing study. Requests to access the datasets should be directed to kslim@kangwon.ac.kr.

## Abbreviations

HSP90	Heat Shock Protein 90
EGFR	Epidermal Growth Factor Receptor
HER2	Human Epidermal Growth Factor Receptor 2
MET	Mesenchymal-Epithelial Transition factor
ALK	Anaplastic Lymphoma Kinase
RAF	Rapidly Accelerated Fibrosarcoma
MEK	Mitogen-Activated Protein Kinase Kinase (MAP2K)
ERK	Extracellular signal-Regulated Kinase
PI3K	Phosphoinositide 3-Kinase
AKT	AKT serine/threonine kinase (Protein Kinase B)
MAPK	Mitogen-Activated Protein Kinase
DNA	Deoxyribonucleic Acid
ATP	Adenosine Triphosphate
DLT	Dose-Limiting Toxicity
ORR	Objective Response Rate
PFS	Progression-Free Survival
HSF-1	Heat Shock Factor 1
IGF-1R	Insulin-like Growth Factor 1 Receptor
MDSCs	Myeloid-Derived Suppressor Cells
NSCLC	Non-Small Cell Lung Cancer
BRAF	v-Raf Murine Sarcoma Viral Oncogene Homolog B
PD-1	Programmed Cell Death Protein 1
PD-L1	Programmed Death-Ligand 1
Tregs	Regulatory T Cells
CDK1	Cyclin-Dependent Kinase 1
CDC25C	Cell Division Cycle 25C
RP2D	Recommended Phase 2 Dose
pCR	Pathological Complete Response
TNBC	Triple-Negative Breast Cancer
dC	Deoxycytidine
dFdCTP	Gemcitabine Triphosphate (active metabolite of gemcitabine)
DDR	DNA Damage Response
Chk1	Checkpoint Kinase 1
DCR	Disease Control Rate
OS	Overall Survival
SD	Stable Disease
PR	Partial Response
5-FU	5-Fluorouracil
CI	combination index
EC_50_	Half Maximal Effective Concentration
DLBCL	Diffuse Large B-Cell Lymphoma
NPC	Nasopharyngeal Carcinoma
RTKs	Receptor Tyrosine Kinases
CBR	Clinical Benefit Rate
MTD	Maximum Tolerated Dose
FOXP3	Forkhead Box P3
CD8^+^	Cluster of Differentiation 8 Positive
HS201-PDT	HS201 Photodynamic Therapy
CXCR3	C-X-C Chemokine Receptor Type 3
CTLA-4	Cytotoxic T-Lymphocyte Associated Protein 4
MD	Molecular Dynamics
ADMET	Absorption
Distribution	Metabolism
Excretion	and Toxicity
AI	Artificial Intelligence
17-AAG	17-N-allylamino-17-demethoxygeldanamycin
17-DMAG	17-dimethylaminoethylamino-17-demethoxygeldanamycin
IGF-1	insulin-like growth factor 1
INF-γ	Interferon-γ
INF-α/β	Interferon-α/β
IL-6	Interleukin 6
FAS ligand	Fas cell surface death receptor ligand
Src	SRC proto-oncogene, non-receptor tyrosine kinase
PDK1	3 Phosphoinositide Dependent Protein Kinase 1
mTORC2	Mechanistic Target Of Rapamycin Complex 2
mTORC1	Mechanistic Target Of Rapamycin Complex 1
IκBα	Inhibitor of Nuclear Factor Kappa B Alpha
IKK	IκB Kinase (complex)
HIF 1α	Hypoxia Inducible Factor 1 Alpha
NF-κB	Nuclear Factor Kappa-Light-Chain-Enhancer of Activated B Cells
SOS	Son of Sevenless (guanine nucleotide exchange factor)
ELK	Ets-Like Kinase (transcription factor, e.g., ELK1)
JAKs	Janus Kinases (JAK1, JAK2, JAK3, TYK2)
TYK2	Tyrosine Kinase 2
BCL-2	B-Cell Lymphoma 2
BAX	Bcl-2-Associated X Protein
Cyt C	Cytochrome C (apoptogenic factor)
Apaf-1	Apoptotic Protease Activating Factor 1
FADD	Fas-Associated Death Domain protein
BCL-XL	B-Cell Lymphoma-Extra Large (also known as BCL2L1)
MCL-1	Myeloid Cell Leukemia-1
BNIP3	BCL2/Adenovirus E1B 19kDa-Interacting Protein 3
c-FLIP	Cellular FLICE-Like Inhibitory Protein
c-Myc	Cellular Myelocytomatosis Oncogene (transcription factor)
Pim-1	Proto-Oncogene Serine/Threonine-Protein Kinase Pim-1
c-FOS	Cellular FBJ Murine Osteosarcoma Oncogene (transcription factor)
EGR1	Early Growth Response Protein 1
PKR	Protein Kinase R (double-stranded RNA-activated kinase)
ISGF3	Interferon-Stimulated Gene Factor 3 (STAT1–STAT2–IRF9 complex)
VEGF-A	Vascular Endothelial Growth Factor A
IL-8 (CXCL8)	Interleukin-8, also known as C-X-C Motif Chemokine Ligand 8
NOS2 (iNOS)	Nitric Oxide Synthase 2, inducible
ADM	Adrenomedullin
IP-10 (CXCL10)	Interferon Gamma-Induced Protein 10, or C-X-C Motif Chemokine Ligand 10
GLUT1	Glucose Transporter 1 (SLC2A1)
LDHA	Lactate Dehydrogenase A
HK2	Hexokinase 2
CA9	Carbonic Anhydrase IX
PFKFB3	6-Phosphofructo-2-Kinase/Fructose-2,6-Bisphosphatase 3
MMP-9	Matrix Metallopeptidase 9 (gelatinase B)
MMP-2	Matrix Metallopeptidase 2 (gelatinase A)
LOX	Lysyl Oxidase
TWIST1	Twist-Related Protein 1 (transcription factor)
FAK	Focal Adhesion Kinase (PTK2)
MMP	Matrix Metallopeptidases (family of proteases)
MIA PaCa-2	Human pancreatic carcinoma cell line (MIA PaCa-2)
PANC-1	Human pancreatic carcinoma cell line (PANC-1)
